# Recovery from hypoxemia and Hypercapnia following noninvasive pressure support ventilation in a patient with statin-associated necrotizing myopathy: a case report

**DOI:** 10.1186/s12890-020-01195-7

**Published:** 2020-06-03

**Authors:** Yuriko Yamamura, Yoshinori Matsumoto, Koh Tadokoro, Yasuyuki Ohta, Kota Sato, Toru Yamashita, Masahiro Yamamura, Ken-Ei Sada, Koji Abe, Jun Wada

**Affiliations:** 1 Department of Nephrology, Rheumatology, Endocrinology and Metabolism, Okayama University Graduate School of Medicine, Dentistry and Pharmaceutical Sciences, Okayama, 700-8558 Japan; 2 Department of Neurology, Okayama University Graduate School of Medicine, Dentistry and Pharmaceutical Sciences, Okayama, 700-8558 Japan; 3 Center for Rheumatology, Okayama Saiseikai General Hospital, Okayama, 700-8511 Japan

**Keywords:** Noninvasive pressure support ventilation, Statin-associated necrotizing myopathy, BIPAP

## Abstract

**Background:**

Statin-associated necrotizing myopathy (SANM) is a rare autoimmune disorder caused by administration of statins. SANM is characterized by weakness due to necrosis and regeneration of myofibers. Here we report the first case of SANM with acute respiratory failure treated with noninvasive pressure support ventilation in addition to immunosuppressants.

**Case presentation:**

A 59-year-old woman who had been treated with 2.5 mg/day of rosuvastatin calcium for 5 years stopped taking the drug 4 months before admission to our hospital due to elevation of creatine kinase (CK). Withdrawal of rosuvastatin for 1 month did not decrease the level of CK, and she was admitted to our hospital due to the development of muscle weakness of her neck and bilateral upper extremities. Anti-3-hydroxy-3-methylglutaryl coenzyme A reductase antibodies were positive. Magnetic resonance imaging showed myositis, and muscle biopsy from the right biceps brachii muscle showed muscle fiber necrosis and regeneration without inflammatory cell infiltration, suggesting SANM. After the diagnosis, she received methylprednisolone pulse therapy (mPSL, 1 g/day × 3 days, twice) and subsequent oral prednisolone therapy (PSL, 30 mg/day for 1 month, 25 mg/day for 1 month and 22.5 mg/day for 1 month), leading to improvement of her muscle weakness. One month after the PSL tapering to 20 mg/day, her muscle weakness deteriorated with oxygen desaturation (SpO2: 93% at room air) due to hypoventilation caused by weakness of respiratory muscles. BIPAP was used for the management of acute respiratory failure in combination with IVIG (20 g/day × 5 days) followed by mPSL pulse therapy (1 g/day × 3 days), oral PSL (30 mg/day × 3 weeks, then tapered to 25 mg/day) and tacrolimus (3 mg/day). Twenty-seven days after the start of BIPAP, she was weaned from BIPAP with improvement of muscle weakness, hypoxemia and hypercapnia. After she achieved remission with improvement of muscle weakness and reduction of serum CK level to a normal level, the dose of oral prednisolone was gradually tapered to 12.5 mg/day without relapse for 3 months.

**Conclusions:**

Our report provides new insights into the role of immunosuppressants and biphasic positive airway pressure for induction of remission in patients with SANM.

## Background

Statin-associated necrotizing myopathy (SANM), characterized by symmetrical weakness and muscle enzyme elevations as a consequence of necrosis and regeneration of myofibers, is a rare disease that occurs in patients treated with statins [1–3]. Previous studies suggested that statins enhance the expression of 3-hydroxy-3-methylglutaryl coenzyme A reductase (HMGCR), the enzymatic target of statins, in genetically susceptible patients, leading to the development of disease-specific antibodies against HMGCR [4–9]. SANM causes life-threatening hypoventilation-mediated hypoxemia and hypercapnia, but therapeutic strategies for SANM have not been established yet.

Noninvasive pressure support ventilation (NIPSV) is the delivery of mechanical ventilation to the lungs without an endotracheal airway by biphasic positive airway pressure (BIPAP) that is applied with two levels of pressure, inspiratory positive airway pressure (IPAP) and expiratory positive airway pressure (EPAP) [10]. The patient’s inspiratory effort triggers the ventilator to deliver a decelerated flow to achieve and maintain a preset pressure, while ventilatory assistance ceases when the patient’s inspiratory flow falls. This modality is useful for patients with hypoxemia and/or hypercapnia due to acute respiratory failure, leading to a reduction of the intubation rate and mortality.

Here we report the first serious case of SANM complicated with hypoxemia and hypercapnia. Combined medications of glucocorticoids, immunosuppressants and intravenous immunoglobulins (IVIG) in addition to ventilatory support with a BIPAP device were required to control disease activity and acute respiratory failure without unexpected side effects including infections.

## Case presentation

**Fig. 1:**
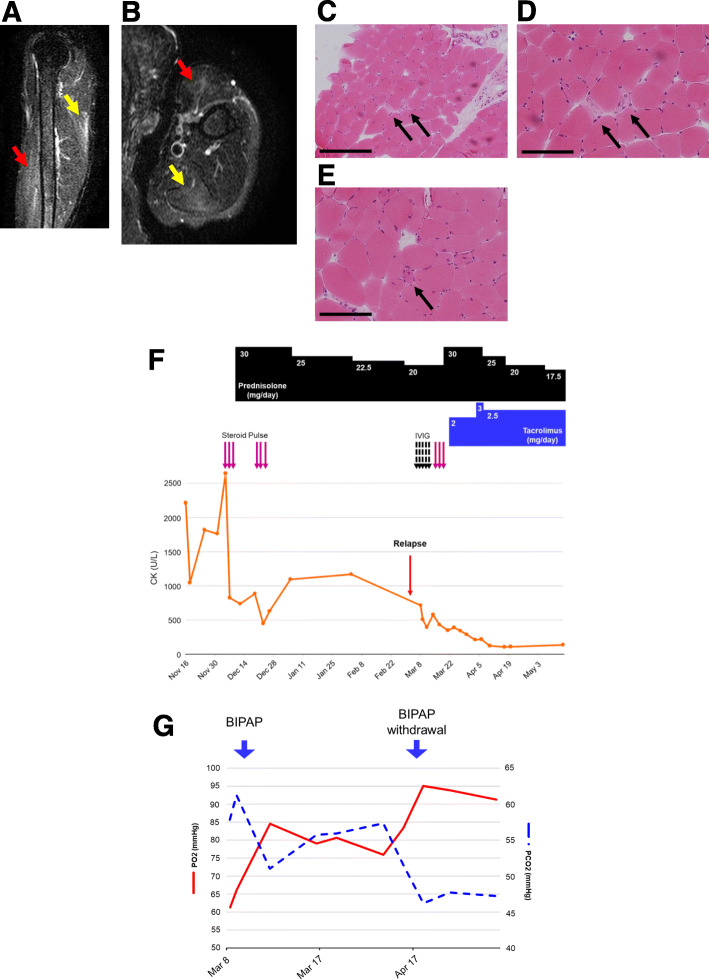
**a** and **b** STIR T2-weighted imaging of MRI in the left proximal upper extremity (**a** sagittal imaging; **b** axial imaging). Red and yellow arrows indicate the left biceps brachii muscle and triceps brachii muscle, respectively. **c** and **d** H&E staining at lower magnification (**c**) and higher magnification (**d**) of muscle biopsy from the right biceps brachii muscle. **e** Another field image of H&E staining of the muscle biopsy from the same muscle. Scale bars: 200 μm (**c**), 100 μm (**d** and **e**). Black arrows indicate a mixture of muscle fiber necrosis and regeneration without inflammatory cell infiltration. **f** Clinical course of the patient showing serum levels of creatine kinase (CK, normal, 41–153 U/L). IVIG: intravenous immunoglobulin. **g** Clinical course of the patient showing severe hypoxia and hypercapnia due to hypoventilation caused by weakness of respiratory muscles. BIPAP was used for the management of acute respiratory failure, which dramatically improved after the commencement of BIPAP support

A 59-year-old woman who had been diagnosed with hyperlipidemia and treated with 2.5 mg/day of rosuvastatin calcium for 5 years stopped taking the drug 4 months before admission to our hospital due to elevation of creatine kinase (CK, 1200 U/L; normal, 41–153 U/L). Withdrawal of rosuvastatin for 1 month did not decrease the level of CK (> 2000 U/L), and she was admitted to our hospital due to the development of muscle weakness of her neck and bilateral upper extremities. Manual muscle testing (MMT) revealed marked muscle weakness of her neck and her paraspinal and bilateral upper extremities (proximal>distal) with normal muscle tone, reflexes but varying power (neck: 4, shoulder abductors: 4/4, elbow flexors: 4/4, elbow extensors: 4/4, wrist extensors: 5/4, hip flexors: 3/3, knee extensors: 5/5, knee flexors: 4/4, ankle plantar flexors: 5/5). There was no evidence of neurological signs or symptoms of the cerebellar and autonomic systems. She had no skin rashes as exemplified by dermatomyositis. Laboratory data showed leukocytosis (10,200/μL; normal, 3300–8600/μL), increased serum levels of muscle enzymes including CK (2212 U/L), myoglobin (Mb, 2030 ng/ml; normal, 18–70 ng/ml) and aldolase (58.1 U/L; normal, 2.7–7.5 U/L) and increased C-reactive protein (CRP) level (1.17 mg/dL; normal, < 0.15 mg/dL) (Table 1). Tests for autoantibodies including antinuclear antibody, rheumatoid factor, anti-Ro/SSA antibody and anti-La/SSB antibody were negative, while disease-specific anti-HMGCR antibodies for necrotizing myopathy were positive (3.9 IU/mL) (Table 1). A computed tomography (CT) scan of the chest, abdomen, and pelvis showed no remarkable findings including infection, interstitial pneumonia and malignancy. Magnetic resonance imaging (MRI) showed high signal intensity on short-TI inversion recovery (STIR) T2-weighted imaging in the left biceps brachii muscle and triceps brachii muscle (Fig. 1a and b). A muscle biopsy from the right biceps brachii muscle showed a mixture of muscle fiber necrosis and regeneration without inflammatory cell infiltration (Fig. 1c-e). These findings suggested the existence of SANM. After admission and diagnosis, she received methylprednisolone pulse therapy (mPSL, 1 g/day × 3 days, twice) and subsequent oral prednisolone therapy (PSL, 30 mg/day) for 1 month, leading to improvement of her muscle weakness with reduction of the serum level of CK (449 U/L) (Fig. 1f). PSL was tapered to 25 mg/day (for 1 month) and 22.5 mg/day (for 1 month) before tapering to 20 mg/day.

**Table 1:**
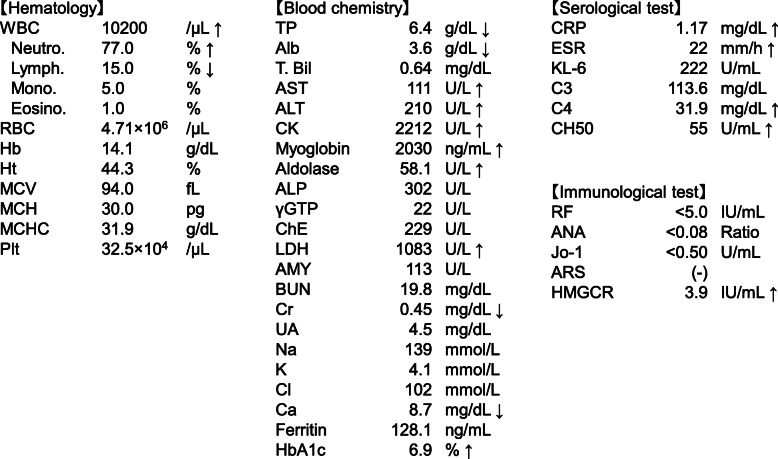
Laboratory data of the patient on admission

*CRP* C-reactive protein, *RF* rheumatoid factor, *ANA* anti-nuclear antibodies, *Jo-1* anti-Jo-1 antibodies, *ARS* anti-aminoacyl-tRNA synthetase antibodies, *HMGCR* anti-3-hydroxy-3-methylglutaryl coenzyme A reductase antibodies

One month after the PSL tapering to 20 mg/day, her muscle weakness deteriorated with elevation of CK level (717 U/L) (Fig. 1f, Relapse) and oxygen desaturation (SpO2: 93% at room air). Arterial blood gas analysis showed severe hypoxia and hypercapnia due to hypoventilation caused by weakness of respiratory muscles, and BIPAP was used for the management of acute respiratory failure in combination with IVIG (20 g/day × 5 days) followed by mPSL pulse therapy (1 g/day × 3 days), oral PSL (30 mg/day × 3 weeks, then tapered to 25 mg/day) and tacrolimus (3 mg/day) (Fig. 1f and g). Twenty-seven days after the start of BIPAP, she was weaned from BIPAP with improvement of muscle weakness, hypoxemia and hypercapnia and reduction of the serum CK level (126 U/L) to a normal level (Fig. 1f and g). After she achieved remission, the dose of oral prednisolone was gradually tapered to 12.5 mg /day without relapse for 3 months.

## Discussion

During the course of the present case, we found that glucocorticoid monotherapy is not sufficient to control disease activity and that NIPSV is useful for the management of hypoxemia and hypercapnia observed in patients with SANM. SANM is classified as an autoimmune-associated myopathy following abnormal production of anti-HMGCR autoantibodies after statin medications, different from well-established polymyositis/dermatomyositis-associated antibodies against aminoacyl-tRNA synthetases (ARS). Statin medications are one of the most common therapeutic strategies for hyperlipidemia to reduce morbidity and mortality for both cardiovascular and cerebral vascular diseases [1], whereas 5–20% of the patients stop taking a statin due to side effects including elevation of serum CK level regardless of the presence or absence of myalgia [11, 12]. While statin-related myopathy is relieved after discontinuation of the statin in most cases, 2 or 3 of 100,000 statin-treated patients develop severe myopathy that displays proximal muscle weakness and/or muscle pain with elevation of CK level. It has been reported that anti-HMGCR antibodies could induce muscle weakness in mice through a complement-mediated mechanism [13]. However, the role of anti-HMGCR autoantibodies in the pathogenesis of SANM has not been clarified yet. Despite the presence of autoantibodies, necrotic and regenerating myofibers without inflammatory infiltrates are predominantly observed in SANM, indicating that the pathogenesis of SANM may be different from that of common inflammatory myopathy, polymyositis/dermatomyositis. Consistent with these findings, glucocorticoid monotherapy, which is initially used for polymyositis/dermatomyositis, is not sufficient to control disease activity, and combination therapy of glucocorticoids and immunosuppressants is required for SANM [8, 14–16]. Other groups recommended triple therapy of glucocorticoids, immunosuppressants and IVIG [5] or rituximab [8] for SANM. Our case demonstrates that treatment with immunosuppressants and IVIG in addition to glucocorticoids is required for SANM patients complicated with hypoxemia and hypercapnia due to acute respiratory failure. Further studies are needed to determine the optimal treatment for patients with SANM.

In addition to the combination therapy, we used NIPSV to control respiratory failure caused by SANM. A previous study showed that half of the patients with proximal myopathy had more than 50% reduction of both inspiratory and expiratory muscle strengths and that hypercapnia was likely to occur when respiratory muscle strength and vital capacity were less than 30% of normal and 55% of the predicted value, respectively [17]. Previously, endotracheal intubation with a ventilator was a major therapeutic strategy in patients with acute respiratory failure who did not respond to conventional treatment such as medications and oxygen. In addition to complications related to the procedure of intubation including local trauma, gastric content aspiration and transient hypotension, intubated patients need complete sedation, resulting in limited communication and further muscle weakness. Moreover, those patients have a higher risk of pneumonia, leading to higher mortality [18]. On the other hand, a previous meta-analysis showed that NIPSV reduces the intubation rate and mortality by 50 and 40%, respectively [18]. Therefore, NIPSV may be recommended to control hypoxemia and hypercapnia during the time before induction of remission and subsequent improvement of respiratory muscles in patients with SANM.

## Conclusion

We reported a severe case of SANM complicated with respiratory failure caused by weakness of respiratory muscles. Our report provides new insights into the role of immunosuppressants and biphasic positive airway pressure for induction of remission in patients with SANM.

## Data Availability

NA

## Abbreviations

HMGCR: 3-hydroxy-3-methylglutaryl coenzyme A reductase
BIPAP: Biphasic positive airway pressure
SANM: Statin-associated necrotizing myopathy
CK: Creatine kinase
NIPSV: Noninvasive pressure support ventilation
IPAP: Inspiratory positive airway pressure
EPAP: Expiratory positive airway pressure
IVIg: Intravenous immunoglobulins
Mb: Myoglobin
CRP: C-reactive protein
CT: Computed tomography
MRI: Magnetic resonance imaging
STIR: Short-TI inversion recovery
ARS: Aminoacyl-tRNA synthetases
